# Supplementary material for: Modeling glioblastoma heterogeneity as a dynamic network of cell states
Source: Mol Syst Biol. 2021 Sep 16;17(9):e10105. doi: 10.15252/msb.202010105 (PMC8444284; doi:10.15252/msb.202010105)
Supplement: Supplementary file 5 — Source Data for Figure 3 [file MSB-17-e10105-s001.zip › Figure3A_sourcedata/GSEA_3065/nosigdb_state1.GseaPreranked.1622718409535/LARSSON_STATE_6.html]

Details for gene set LARSSON\_STATE\_6[GSEA]

|  || Dataset | state1 |
| Phenotype | NoPhenotypeAvailable |
| Upregulated in class | na\_neg |
| GeneSet | LARSSON\_STATE\_6 |
| Enrichment Score (ES) | -0.6755288 |
| Normalized Enrichment Score (NES) | -2.3880148 |
| Nominal p-value | 0.0 |
| FDR q-value | 0.0 |
| FWER p-Value | 0.0 |
Table: GSEA Results Summary

  

Fig 1: Enrichment plot: LARSSON\_STATE\_6      
 Profile of the Running ES Score & Positions of GeneSet Members on the Rank Ordered List

  

| PROBE | GENE SYMBOL | GENE\_TITLE | RANK IN GENE LIST | RANK METRIC SCORE | RUNNING ES | CORE ENRICHMENT || 1 | LGALS3 |  |  | 117 | 0.348 | 0.0040 | No |
| 2 | FTL |  |  | 134 | 0.333 | 0.0176 | No |
| 3 | TPT1 |  |  | 220 | 0.287 | 0.0221 | No |
| 4 | ATP6V1F |  |  | 560 | 0.193 | -0.0037 | No |
| 5 | SERF2 |  |  | 695 | 0.173 | -0.0095 | No |
| 6 | CCNI |  |  | 749 | 0.166 | -0.0073 | No |
| 7 | PHPT1 |  |  | 890 | 0.146 | -0.0149 | No |
| 8 | OST4 |  |  | 932 | 0.142 | -0.0126 | No |
| 9 | RPL34 |  |  | 1023 | 0.131 | -0.0158 | No |
| 10 | PTMS |  |  | 1066 | 0.126 | -0.0143 | No |
| 11 | HNRNPDL |  |  | 1086 | 0.124 | -0.0106 | No |
| 12 | C1orf122 |  |  | 1175 | 0.115 | -0.0143 | No |
| 13 | TNFRSF21 |  |  | 1222 | 0.110 | -0.0140 | No |
| 14 | FTH1 |  |  | 1296 | 0.104 | -0.0167 | No |
| 15 | C9orf16 |  |  | 1329 | 0.101 | -0.0153 | No |
| 16 | RPS27 |  |  | 1369 | 0.098 | -0.0148 | No |
| 17 | SRP14 |  |  | 1445 | 0.093 | -0.0182 | No |
| 18 | PFN2 |  |  | 1462 | 0.091 | -0.0157 | No |
| 19 | FABP5 |  |  | 1470 | 0.091 | -0.0122 | No |
| 20 | SNX3 |  |  | 1585 | 0.083 | -0.0201 | No |
| 21 | FKBP8 |  |  | 1695 | 0.076 | -0.0277 | No |
| 22 | COMMD6 |  |  | 1699 | 0.076 | -0.0246 | No |
| 23 | GABARAPL2 |  |  | 1782 | 0.071 | -0.0297 | No |
| 24 | ATP6V1G1 |  |  | 1946 | 0.063 | -0.0435 | No |
| 25 | SKP1 |  |  | 1999 | 0.060 | -0.0461 | No |
| 26 | BEX1 |  |  | 2118 | 0.054 | -0.0557 | No |
| 27 | SMDT1 |  |  | 2268 | 0.048 | -0.0687 | No |
| 28 | GNAI2 |  |  | 2281 | 0.048 | -0.0677 | No |
| 29 | LAMTOR4 |  |  | 2553 | 0.038 | -0.0937 | No |
| 30 | RPS11 |  |  | 2781 | 0.031 | -0.1155 | No |
| 31 | PFDN5 |  |  | 2857 | 0.029 | -0.1218 | No |
| 32 | TOMM7 |  |  | 2940 | 0.026 | -0.1290 | No |
| 33 | SCP2 |  |  | 3047 | 0.024 | -0.1388 | No |
| 34 | BRI3 |  |  | 3915 | 0.006 | -0.2271 | No |
| 35 | C4orf3 |  |  | 4151 | 0.002 | -0.2511 | No |
| 36 | MARCH2 |  |  | 5234 | -0.016 | -0.3610 | No |
| 37 | COL4A3BP |  |  | 5776 | -0.025 | -0.4152 | No |
| 38 | HNRNPA2B1 |  |  | 6142 | -0.032 | -0.4511 | No |
| 39 | ZNF428 |  |  | 6155 | -0.032 | -0.4508 | No |
| 40 | BEX3 |  |  | 6478 | -0.038 | -0.4820 | No |
| 41 | MAP1LC3A |  |  | 6511 | -0.039 | -0.4835 | No |
| 42 | EEF1A1 |  |  | 6584 | -0.040 | -0.4890 | No |
| 43 | ID1 |  |  | 6749 | -0.044 | -0.5038 | No |
| 44 | FAM20C |  |  | 6805 | -0.045 | -0.5073 | No |
| 45 | MARCKSL1 |  |  | 6861 | -0.046 | -0.5108 | No |
| 46 | RAB31 |  |  | 7256 | -0.057 | -0.5485 | No |
| 47 | RTL8C |  |  | 7586 | -0.066 | -0.5792 | No |
| 48 | HOPX |  |  | 7850 | -0.075 | -0.6026 | No |
| 49 | TUBA1A |  |  | 7955 | -0.080 | -0.6096 | No |
| 50 | PNRC1 |  |  | 8006 | -0.081 | -0.6110 | No |
| 51 | MXD4 |  |  | 8080 | -0.084 | -0.6146 | No |
| 52 | ID2 |  |  | 8360 | -0.097 | -0.6386 | No |
| 53 | SH3BGRL |  |  | 8627 | -0.113 | -0.6607 | No |
| 54 | SESN3 |  |  | 8632 | -0.113 | -0.6559 | No |
| 55 | CTNNA1 |  |  | 8686 | -0.116 | -0.6560 | No |
| 56 | BNIP3L |  |  | 8707 | -0.118 | -0.6526 | No |
| 57 | FEZ1 |  |  | 8932 | -0.137 | -0.6692 | Yes |
| 58 | MT3 |  |  | 8954 | -0.139 | -0.6650 | Yes |
| 59 | TTC3 |  |  | 9024 | -0.144 | -0.6655 | Yes |
| 60 | PBX1 |  |  | 9047 | -0.147 | -0.6610 | Yes |
| 61 | CTXN1 |  |  | 9072 | -0.150 | -0.6566 | Yes |
| 62 | HEY1 |  |  | 9215 | -0.168 | -0.6634 | Yes |
| 63 | ZMAT3 |  |  | 9300 | -0.181 | -0.6637 | Yes |
| 64 | METRN |  |  | 9381 | -0.196 | -0.6629 | Yes |
| 65 | FHL1 |  |  | 9398 | -0.200 | -0.6553 | Yes |
| 66 | RND2 |  |  | 9432 | -0.208 | -0.6492 | Yes |
| 67 | MAGED1 |  |  | 9440 | -0.209 | -0.6403 | Yes |
| 68 | PLEKHB1 |  |  | 9466 | -0.218 | -0.6329 | Yes |
| 69 | FOXO3 |  |  | 9503 | -0.226 | -0.6262 | Yes |
| 70 | CITED1 |  |  | 9535 | -0.234 | -0.6186 | Yes |
| 71 | EFTUD2 |  |  | 9545 | -0.237 | -0.6087 | Yes |
| 72 | DKK3 |  |  | 9558 | -0.244 | -0.5987 | Yes |
| 73 | NFIB |  |  | 9572 | -0.249 | -0.5886 | Yes |
| 74 | FYN |  |  | 9575 | -0.250 | -0.5774 | Yes |
| 75 | TXNIP |  |  | 9639 | -0.276 | -0.5712 | Yes |
| 76 | IRS2 |  |  | 9641 | -0.277 | -0.5586 | Yes |
| 77 | TUBB2A |  |  | 9669 | -0.293 | -0.5479 | Yes |
| 78 | CEBPD |  |  | 9675 | -0.295 | -0.5348 | Yes |
| 79 | TSC22D4 |  |  | 9677 | -0.296 | -0.5214 | Yes |
| 80 | SOX4 |  |  | 9704 | -0.318 | -0.5094 | Yes |
| 81 | GABBR2 |  |  | 9707 | -0.323 | -0.4948 | Yes |
| 82 | CRYAB |  |  | 9722 | -0.333 | -0.4810 | Yes |
| 83 | SOX9 |  |  | 9754 | -0.358 | -0.4677 | Yes |
| 84 | OLFM2 |  |  | 9760 | -0.365 | -0.4515 | Yes |
| 85 | NLRP1 |  |  | 9761 | -0.365 | -0.4348 | Yes |
| 86 | MARCKS |  |  | 9763 | -0.370 | -0.4179 | Yes |
| 87 | CD74 |  |  | 9766 | -0.376 | -0.4009 | Yes |
| 88 | C4orf48 |  |  | 9774 | -0.384 | -0.3840 | Yes |
| 89 | ID3 |  |  | 9778 | -0.389 | -0.3664 | Yes |
| 90 | CLU |  |  | 9795 | -0.418 | -0.3489 | Yes |
| 91 | SPARCL1 |  |  | 9798 | -0.423 | -0.3297 | Yes |
| 92 | QKI |  |  | 9803 | -0.430 | -0.3104 | Yes |
| 93 | SAT1 |  |  | 9807 | -0.445 | -0.2903 | Yes |
| 94 | SEPT7 |  |  | 9821 | -0.500 | -0.2687 | Yes |
| 95 | S100B |  |  | 9822 | -0.507 | -0.2454 | Yes |
| 96 | FABP7 |  |  | 9825 | -0.513 | -0.2221 | Yes |
| 97 | TUBB2B |  |  | 9864 | -0.809 | -0.1889 | Yes |
| 98 | NEAT1 |  |  | 9866 | -0.816 | -0.1516 | Yes |
| 99 | PMP2 |  |  | 9871 | -0.982 | -0.1069 | Yes |
| 100 | C1QL1 |  |  | 9876 | -1.171 | -0.0536 | Yes |
| 101 | GFAP |  |  | 9877 | -1.174 | 0.0002 | Yes |
Table: GSEA details [plain text format]

  

Fig 2: LARSSON\_STATE\_6: Random ES distribution      
 Gene set null distribution of ES for **LARSSON\_STATE\_6**
